# Lysyl Oxidase (LOX): Functional Contributions to Signaling Pathways

**DOI:** 10.3390/biom10081093

**Published:** 2020-07-22

**Authors:** Rozalia Laczko, Katalin Csiszar

**Affiliations:** Department of Quantitative Health Sciences, John A. Burns School of Medicine, University of Hawaii, 651 Ilalo Street, Honolulu, HI 96813, USA; Rozalia@hawaii.edu

**Keywords:** lysyl oxidase (LOX), epidermal growth factor receptor (EGFR), platelet derived growth factor (PDGF), vascular endothelial growth factor (VEGF), transforming growth factor β (TGF-β), integrins, inflammation, steroid signaling

## Abstract

Cu-dependent lysyl oxidase (LOX) plays a catalytic activity-related, primary role in the assembly of the extracellular matrix (ECM), a dynamic structural and regulatory framework which is essential for cell fate, differentiation and communication during development, tissue maintenance and repair. LOX, additionally, plays both activity-dependent and independent extracellular, intracellular and nuclear roles that fulfill significant functions in normal tissues, and contribute to vascular, cardiac, pulmonary, dermal, placenta, diaphragm, kidney and pelvic floor disorders. LOX activities have also been recognized in glioblastoma, diabetic neovascularization, osteogenic differentiation, bone matrix formation, ligament remodeling, polycystic ovary syndrome, fetal membrane rupture and tumor progression and metastasis. In an inflammatory context, LOX plays a role in diminishing pluripotent mesenchymal cell pools which are relevant to the pathology of diabetes, osteoporosis and rheumatoid arthritis. Most of these conditions involve mechanisms with complex cell and tissue type-specific interactions of LOX with signaling pathways, not only as a regulatory target, but also as an active player, including LOX-mediated alterations of cell surface receptor functions and mutual regulatory activities within signaling loops. In this review, we aim to provide insight into the diverse ways in which LOX participates in signaling events, and explore the mechanistic details and functional significance of the regulatory and cross-regulatory interactions of LOX with the EGFR, PDGF, VEGF, TGF-β, mechano-transduction, inflammatory and steroid signaling pathways.

## 1. Introduction

Cu-dependent lysyl oxidase (LOX), one of five members of the LOX family, contributes to functions of the extracellular matrix (ECM) by promoting the formation of intra- and inter- molecular cross-linkages of ECM components and the development of tissue-specific insoluble matrices [1,2]. The ECM provides a local structural and signaling environment that controls cell fate, adhesion, proliferation, differentiation, migration and communication during development, tissue maintenance and repair, and various pathological processes. In addition to its activity in the ECM of skin, lung, cardiovascular, epithelial and other tissues, LOX has also been recognized as playing a significant role in cellular and nuclear functions.

The LOX gene is tightly regulated during development and aging and has been noted for its aberrant expression patterns in a range of disorders. The activation of the LOX protein is critically dependent on Cu concentrations and redistribution involving the Cu-uptake transporter-1 (CTR1) and the Cu-efflux pump ATP7A. In the ECM, the pro-LOX is proteolytically activated by procollagen C-proteinases [3] into a 30 kDa enzyme and an 18 kDa N-terminal pro-peptide fragment (LOX-PP). The LOX-PP has a range of well-characterized functions which are independent of LOX. These activities, primarily associated with carcinogenesis, are beyond the focus area of the present manuscript, but were the subject of a recent, comprehensive review [4].

Extracellular matrix processed LOX can reenter the cytoplasm to associate with the cytoskeleton, and has been localized in the nuclei in different cell types, wherein, it was proposed to modify the chromatin structure in interactions with histones as part of epigenetic regulatory mechanisms [5,6]. The internalization and translocation of LOX into the nucleus appears to involve nuclear localization sequences, but the mechanistic details of the nuclear import of LOX remain only partly characterized [7]. 

A recent three dimensional (3-D) model of LOX generated by homology modeling recaptured the essential domains, the coordination of the Cu ion, the lysyl tyrosylquinone LTQ cofactor, disulfide bridges and the catalytic site within a groove surrounded by two fluctuating loops with variable openings which are suitable for the accommodation of LOX substances of different sizes [8]. LOX also participates in various activity-independent functions, the mechanistic details of which remain to be fully characterized. 

Altered LOX expression and functions have been associated with vascular, cardiac, pulmonary, dermal, placenta, diaphragm, kidney and pelvic floor disorders, glioblastoma, diabetic neovascularization, osteogenic differentiation and bone matrix formation, ligament remodeling, polycystic ovary syndrome, fetal membrane rupture and stages of tumor progression and metastasis in various cancer types. Many of these processes involve interactions of LOX with signaling pathways including the epidermal growth factor receptor (EGFR), platelet derived growth factor (PDGF), vascular endothelial growth factor (VEGF), transforming growth factor β (TGF-β), ERK, NF-κB, PI3K/AKT, SMAD, MAPK, FAK/AKT, inflammatory and steroid regulatory pathways. 

In this review, we aim to explore the involvement of LOX in major signaling events, including the relationships and/or mechanistic elements and functional significance of regulatory and cross-regulatory LOX interactions. Secondary signaling mechanisms due to peroxide generated during the catalytic reaction of LOX, including HIF-1α, and the involvement of LOX in various tumor types, have been reviewed extensively and are not discussed here. Tumor cell-related functions of LOX have emerged, however, within signaling frameworks, and brief summaries are provided in the relevant contexts. 

## 2. Caveolae Compartmentalized LOX: Angiotensin II and Epidermal Growth Factor Receptor Cross-Regulation

In a murine model of angiotensin II (Ang II)-induced abdominal aortic aneurysm (AAA), genome-wide transcriptional profiling studies identified LOX, together with ADAM17 and epidermal growth factor receptor (EGFR), as highly ranked gene subnetworks [9], and localized LOX within vascular smooth muscle cell (VSCM) caveolae [10]. The membrane microdomain caveolae facilitate the temporal and spatial coordination of signaling events, including those promoted by Ang II characterized in both VSMC and endothelial cells. Caveolae have been linked to cardiovascular diseases including atherosclerosis, dyslipidemia, cardiac fibrosis, insulin resistance, inflammation, oxidative stress and AAA, many of which also involve LOX. While many of the specific details of LOX functioning in these contexts remain to be determined, those relevant to EGFR provide some degree of insight, as detailed below.

Caveolae-compartmentalized and Ang II-induced signals involve metalloprotease ADAM17/tissue necrosis factor-α (TNF-α), converting the enzyme responsible for epidermal growth factor receptor (EGFR) transactivation, as analyzed in VSMC/ADAM17-deficienct mice [11]. The activated EGFR is known to regulate LOX expression via the PI3K/AKT, MEK/ERK and SAPK/JNK pathways, as observed in human non-small cell lung carcinoma cell lines and confirmed in vivo in an orthotopic metastasis mouse model and in human tumor tissue microarray analysis [12]. In turn, LOX regulation of EGFR also occurs, and was found, in primary and metastatic breast cancer cells, to be linked to the suppression of TGFβ1 signaling with the involvement of HTRA1, leading to increased expression of the EGF-like domain-containing MATN2 that traps EGFR at the cell surface, as part of its activation by EGF [13].

Ang II-induced and catalytically active (responsive to β-aminopropionitrile (BAPN) inhibition) LOX was also reported to contribute to vascular stiffening and arterial hypertension in vivo in mice [14], and to accelerate cardiac remodeling and hypertrophy in a mouse model with elevated expression of LOX in cardiomyocytes and cardiofibroblasts [15]. The Ang II-stimulated hypertensive mice overexpressing active LOX accompanied by increased H_2_O_2_ (the byproduct of the catalytic reaction of LOX with its substrates) showed both increased vascular stiffness and oxidative stress that promoted p38MAPK activation as part of the signaling pathway involved in hypertension-associated vascular remodeling [16].

The contribution of Ang II-associated signals to EGFR transactivation that target LOX is supported by several mouse models, human carcinoma cell lines and human tumor tissue relevant to the pathomechanisms of AAA, vascular remodeling and hypertension, and to primary and metastatic lung carcinoma. Reverse LOX-mediated regulation of EGFR cell surface availability appears to be specific to primary and metastatic breast cancer cell lines.

## 3. LOX in Platelet Derived Growth Factor Signaling

Among other functions, a role of catalytically-active LOX has been identified in platelet-derived growth factor (PDGF) signaling through the modification/oxidation of lysyl residues of the cell surface PDGF receptor-β (PDGFRβ) with modified (faster) turnover of the phosphorylated elements of the PDGFR-dependent regulatory pathway, including SHP2, AKT1 and ERK1/2, a process characterized during in vitro chemotactic response in rat aortic smooth muscle cells and mouse embryonic fibroblasts [17,18]. In a network analysis of low sheer stress-induced vascular remodeling of rat aorta focused on endothelial and vascular smooth muscle cell interactions, increased LOX was found in parallel to increases of PDGF, TGF-β1 and phosphorylated ERK1/2 [19]. 

Increased LOX was found to contribute to myelofibrosis in bone marrow biopsies of patients with myeloproliferative neoplasms [20], and the link between LOX and PDGF was further explored. During megakaryocyte proliferation and differentiation, enzymatically-active LOX is highly expressed in the early stage of expansion and differentiation, and is involved in optimal PDGF signaling and cell proliferation. In later differentiation stages, LOX activity subsides, slowing proliferation and allowing differentiation of megakaryocytes into platelets, as confirmed and experimentally recaptured under BAPN inhibitory conditions in GATA-1(low) mice, where inhibition of LOX activity, via the reduction of PDGFRβ binding to cells, also reduced PDGF signaling [21]. 

LOX was also shown to be essential in endothelial cells in vitro and in angiogenesis in vivo by activating AKT through PDGFRβ stimulation, resulting in increased VEGF expression, a mechanism characterized in models of both colorectal cancer and breast cancer, and supported by clinical correlations between LOX, VEGF levels and blood vessel formation in colorectal tumors [22]. 

The role of LOX in optimizing PDGF signaling is further supported by studies of a LOX paralogue, the enzymatically active LOXL2, that, in an orthotopic oral cancer mouse model and human gingival fibroblasts, provided direct evidence for the oxidation of PDGFRβ promoting oral fibrosis and oral cancer [23,24].

Platelet derived growth factor signaling is not only modulated by active LOX, but has been reported to also control LOX. In human periodontal, ligament-derived, mesenchymal stem cells, PDGF treatment stimulated cell proliferation and induced a significant increase in LOX activity [25]. The details of the role of PDGF in controlling LOX activity, and whether this interaction is more general or limited to these stem cells, remain unclear.

Consistent in vitro and in vivo evidence demonstrated in various cell types and animal models, and supported by data with LOXL2, collectively confirm the role of LOX in modifying PDGF signaling by oxidizing the lysyl residues of the cell surface receptor PDGFRβ, thereby contributing both to the regulation of megakaryocyte expansion and differentiation, and to vascular remodeling and angiogenesis. 

## 4. LOX and Vascular Endothelial Growth Factor

Mutual regulatory activities exist between LOX and vascular endothelial growth factor (VEGF). During angiogenesis in stimulated endothelial cells, LOX was shown to activate AKT via PDGFRβ, resulting in enhanced VEGF expression. Furthermore, LOX-promoted angiogenesis was diminished through inhibition of PDGFRβ, AKT and VEGF signaling [22]. Positive correlation of LOX and VEGF expression levels, dose-dependent upregulation of both by TGF-β, and under siRNA LOX conditions, decreased VEGF and p38 MAP signaling were noted in hepatocellular carcinoma cells [26]. In tumor tissues of hepatocellular carcinoma, upregulated LOX similarly correlated with increased VEGF and reduced overall survival related to increased tumor cell proliferation, migration and invasion [24]. In proliferative diabetic retinopathy, neovascularization and basement membrane changes are enhanced with increased glucose concentration and time exposure. The diabetic neovascular membrane was found to have increased LOX immunostaining, in spite of reduced LOX protein and activity, both of which were effectively restored by VEGF in ARPE cells [27].

Further evidence was reported for LOX in mediating VEGF-induced in vitro angiogenesis and osteogenic differentiation of human dental pulp cells. Exogenic VEGF significantly upregulated LOX mRNA and activity, together with an increase of angiogenic mRNAs and capillary tube formation. In contrast, LOX gene silencing and BAPN inhibition of LOX activity diminished the angiogenic effects of VEGF. Under these conditions, VEGF-induced phosphorylation of AKT, ERK, JNK and p38, and activation of NF-κB were inhibited by LOX silencing and BAPN [28]. Beta-aminopropionitrile (BAPN) inhibition of LOX activity, resulting in decreased phosphorylation of AKT and MAPK, was also noted during umbilical vein endothelial cell angiogenesis [29]. In a highly tumorigenic subpopulation of hepatocellular carcinoma containing tumor-initiating, sphere-forming cells that generate tumors with vascular enrichment, the upregulation of LOX correlated with an increase in secreted VEGF that promoted tube formation of endothelial cells, an effect that was blocked by BAPN, demonstrating the pivotal role of LOX in these cells [30]. 

The VEGF axis and LOX in HIF-1α-driven solid tumor angiogenesis showed branching [31] of the activated pathways that also induced each other [22,27]. There is a direct positive regulatory loop linking LOX and HIF-1α. LOX activated by HIF-1α can further induce HIF-1α through PI3K/AKT signaling, a process that involves LOX-generated H_2_O_2_ promoting colorectal carcinoma cell proliferation and in vivo tumor growth [32]. 

Other mechanisms by which LOX and VEGF are coregulated are Cu-related and involve HIF-1α and HDAC2. The chaperone function of the Cu-transport protein Antioxidant-1 (Atox-1) is an important contributor to VEGF-induced angiogenesis that involves LOX activation, as demonstrated in Atox1-defificent mice [33]. VEGF and LOX are also coregulated by the Cu-dependent activation of HIF-1α, the absence of which contributes to emphysema (shown in rat lungs) and involves histone deacetylase HDAC2-mediated expression of HIF-1α, VEGF [34]. 

In conclusion, there is positive coregulation of LOX and VEGF expression controlled by TGF-β in hepatocellular carcinoma cells and tumors that correlates with reduced overall survival. The coordinated upregulation of both LOX and VEGF also occurs in in vitro angiogenesis and in vivo in neovascularization in diabetic retinopathy. The Cu-dependent coregulation of VEGF and LOX plays roles in angiogenesis and in emphysema. VEGF and LOX can, furthermore, regulate each other, although this finding has only been observed in in vitro angiogenesis. 

## 5. Cross-Regulation of Transforming Growth Factor β and LOX

Active LOX has strong associations with lung, arterial, cardiac, dermal and kidney fibrosis, and with cellular fibrotic mechanisms including platelet-induced epithelial-mesenchymal transition and fibroblast to myofibroblast transdifferentiation, specifically in wounds characterized by injury and repair cycles. In the fibrotic process, transforming growth factor-β1 (TGF-β1)/Smad3 signaling drives significant upregulation of the LOX mRNA, protein levels, and activity in fibroblasts, osteoblasts, epithelial and aortic smooth muscle cells. In an in vivo TGF-β-induced synovial fibrosis model, and in end-stage human osteoarthritis, TGF-β upregulated LOX expression was confirmed [35]. Increased TGF-β and TGF-βR1 contributed to the maintenance of symptoms; however, TGF-β and TGF-βR1 induction of LOX was limited to the initial stages of capsule deformation noted in shoulder instability [36]. 

LOX and the type I A1 and A2 collagen genes (COL1A1/A2) have TGF-β response promoter elements that ensure their coregulated expression [37]. Consequently, TGF-β/p38 mitogen-activated protein kinase (p38MAPK) signaling promotes both LOX levels and collagen synthesis characterized in obesity-associated, fetal muscle fibrogenesis. In adult rat cardiac fibroblasts, TGF-β1 upregulation of LOX mRNA, protein and activity (accompanied by increases in collagen type I, III and BMP-1 expression) involved PI3K, Smad3, p38-MAPK, JNK and ERK1/2, and indicated the merging of the PI3K/Akt and Smad pathways [38]. LOX upregulation by TGF-β/Smad2/3 signaling was shown to promote myocardial fibrosis and chronic heart failure. In this rat model, TGF-β/Smad2/3 promoted the expression of C-jun, an AP-1 transcription factor subunit that induced LOX gene expression. Furthermore, LOX inhibition by BAPN was shown to attenuate TGF-β-mediated downstream effects, including cardiac dysfunction, ventricular remodeling and myocardial fibrosis [39]. 

The involvement of the TGF-β /SMAD3 pathway in regulating LOX expression was also confirmed in preeclampsia, a pregnancy-specific condition, in which decreased LOX was shown to be in close association with impaired trophoblast invasion in preeclamptic placenta. In testing trophoblast cells, knockdown of LOX suppressed cell migration and invasion with activation of the TGF-β /SMAD3 pathway. This effect could be rescued by inhibiting the TGF-β/SMAD3 pathways, known to be active not only in cells, but also in clinical samples [40]. 

In skeletal growth, SMAD4, a central element of the TGF-β/BMP pathway, controls LOX. Mice lacking Smad4 present a combination of phenotypic features which are typical in osteogenesis imperfecta, cleidocranial dysplasia and Wnt deficiency syndromes. In this model, osteoblasts fully differentiated, but the lack of Smad4- and Runx2-regulated Lox resulted in a disorganized and hypomineralized collagen matrix [41].

TGF-β is a central determinant of the cellular response to hypoxia. Coordinated upregulation of LOX and TGF-β, both at the protein and mRNA levels, have been shown to be induced in vitro and in vivo by hypoxia, and downregulated by hypercapnia in a rat model of hypoxic pulmonary hypertension [42].

A LOX/TGF-β feedback loop, characterized in skeletal muscle development, was shown to play a role in maintaining balance between muscle components. In a mouse model, myofiber-derived, secreted LOX was able to control the balance between myofibers and muscle connective tissue by modulating TGF-β1 signaling, thereby inhibiting myofiber differentiation and enhanced muscle connective tissue development [43]. LOX modulation of TGF-β signaling also occurs in idiopathic pulmonary fibrosis. In the inflammatory stage, the inhibition of LOX manifests in diminished inflammatory cell infiltration, TGF-β signaling and myofibroblast accumulation, mechanisms that are not active in the fibrogenic phase. Conversely, ectopic LOX sensitizes fibrosis-resistant mice to inflammation and bleomycin-induced lung fibrosis and is critical for the progression of lung fibrosis by enhancing the inflammatory response [44]. 

A notable direct interaction occurs between active LOX and TGF-β1, through which LOX can reduce TGF-β1-stimulated SMAD3 phosphorylation. While this effect depends on the amine oxidase activity of LOX, it does not involve peroxide-related mechanisms. In this direct interaction, LOX was proposed, but not proven experimentally, to be responsible for oxidative deamination of lysine residues in TGF-β1, either by changing charges or by covalently stabilizing TGF-β1 conformation. Both of these changes may reduce the efficiency of receptor binding of TGF-β1 and result in reduced signaling via SMAD3 in a cross-talk with phosphoinositide 3-kinase (PI3K) and AKT serine-threonine protein kinase [45].

There is consistent data to support TGF- β1/Smad3 signaling-driven LOX induction resulting in cellular fibrotic mechanism, multiple tissue fibrosis, ventricular remodeling and impaired trophoblast invasion in preeclampsia. LOX can also modulate TGF-β via a feedback loop which is active in skeletal muscle development and idiopathic pulmonary fibrosis. Additionally, LOX in direct interaction with TGF-β1 may reduce TGF-β1-stimulated SMAD3 activation.

## 6. LOX in Integrin-Mediated Mechano-Transduction

Cell-ECM interactions involve reciprocal forces that are prominently mediated and translated into intracellular signals by integrins [46], and contribute to the mechano-regulation of LOX with distinctly different control of LOX expression by low- and high-level mechanical forces. High-level stretching does not affect LOX activity, while low-level stretching enhances LOX gene expression and activity, that, together with increased expression of COL1A1 and COL3A1 genes, promote ECM stabilization, as demonstrated in human periodontal ligament cells [47]. The physical properties of the cellular microenvironment influence LOX expression via interactions of integrin α2β1 with collagen type I, as seen in cardiac fibroblasts [48]. Type I collagen (COL1) is an abundant scaffolding protein stabilized by LOX cross-linking that promotes the formation of integrin complexes, leading to the activation of the TGF-β pathway and further increase in LOX expression [49]. These pathways have also been recognized as playing significant roles within the tumor microenvironment [50]. A tissue microarray analysis of genes associated with increased stromal stiffness confirmed a relationship between elevated levels of LOX and COL1 expression involving integrin β1/GSK-3β/β-catenin signaling associated with hepatocellular carcinoma development and progression [51].

ECM stiffness promotes inflammatory activation in lung endothelial cells, a process that involves LOX activation and enhanced expression of the microtubule-associated Rho A activator GEF-H1, and that can be reversed by LOX suppression, resulting in diminished GEF-H1 and inflammation control [52]. LOX is also involved in the matrix-mediated mechanotransduction observed in posttraumatic osteoarthritis. This process involves age- and mechanical stress-induced increases in matrix stiffness, and destabilized chondrocyte catabolism and anabolism via the Rho-Rho kinase myosin light chain axis [53]. In an organotypic skin culture model of recessive dystrophic epidermolysis bullosa characterized by high risk for early cutaneous squamous cell carcinoma, injury-driven modifications of the microenvironment, including increased TFG-β, ECM cross-linking and tissue stiffening, the prominent integrin β1/pFAK/pAKT mechano-signaling proved to be both TGF-β- and LOX-dependent [54]. 

Elements of regulatory interactions involving LOX-mediated matrix stiffness were also revealed in hepatocellular carcinoma. The gradual upregulation of LOX and VEGF resulted in increased matrix rigidity that promoted angiogenesis with integrin β1 as the major mediator activating the PI3K/AKT pathway [55]. 

LOX was also demonstrated to function as a macrophage chemoattractant involving the integrin β1/PYK2 pathway to promote macrophage infiltration and, in heterotypic cell interactions, glioma cell survival and angiogenesis. The results from this study also revealed YAP1 regulation of LOX expression via SRC/AKT/YAP1. The transcriptional coactivator YAP1 functions together with TEAD transcription factors through distal enhancers involving H3K27 acetylation (H3K27ac). Specific binding of YAP1 and H3K27ac to the LOX promoter was confirmed in glioblastoma cell models. Furthermore, SRC/AKT/YAP1 and YAP1/LOX signaling were shown to positively correlate with macrophage density and reduced overall survival in glioblastoma multiforme patients [56]. The involvement of LOX, not only as a target, but as a modulator of YAP1, was reported in unilateral ureteral obstruction, where upregulation of the ERK/YAP1/Kfl5/cyclin D axis was suppressed by LOX inhibition [57].

Related to both mechano-transduction and inflammation, the mechano growth factor-E (MGF-E) in osteoarthritic fibroblast-like synoviocytes was reported to upregulate LOX mRNA, concomitant with downregulated TNF-α and interleukin-1 beta (IL-1β), resulting in mitigated inflammation [58]. Table 1 provides a summary of LOX interactions with, and contribution to, signaling mechanisms, including the regulatory elements involved and the cell, tissue types, model systems and/or pathological condition in which these interactions were identified or characterized.

In conclusion, LOX/integrin-associated interactions include a LOX/integrin/TGF-β/LOX feedback loop, integrin β1/pFAK/pAKT mechano-signaling (that is both TGF-β and LOX dependent), the integrin β1/PYK2 pathway, LOX regulation though SRC/AKT/YAP1 and a proposed LOX-mediated modulation of YAP1. These processes contribute to matrix stiffness-driven epithelial cell activation, angiogenesis, macrophage infiltration and glioma cell survival.

## 7. LOX in Inflammatory Pathways

Chronic TNF-α-induced inflammation is a feature of lung diseases with an inflammatory component, including asthma, emphysema, fibrosis and chronic obstructive pulmonary disease. The inflammatory process is characterized by TNF-α-dependent Cu deficiency, leading to the downregulation of LOX, and is accompanied by decreased expression of Vegf and Fak, as reported in a mouse model [59]. Additionally, TNF-α regulates LOX through diverse cell- and tissue-specific transcriptional and posttranscriptional mechanisms. 

In pluripotent mesenchymal cells, TNF-α was shown to downregulate LOX, independent of the Wnt3a signaling that is required for osteoblast development. In LOX-overexpressing C3H10T1/2 cells, TNF-α-mediated LOX downregulation occurred via a posttranscriptional mechanism involving miR203. Silencing of LOX in these cells resulted in suppressed growth and osteoblast differentiation, supporting the conclusion that interference with LOX expression in inflammatory conditions such as diabetes, osteoporosis and rheumatoid arthritis may result in a diminishing pluripotent cell pool, a condition known to also contribute to osteopenia [60]. Furthermore, TNF-α modulates/decreases vascular LOX expression and activity that, in endothelial cells, occurs through a transcriptional mechanism involving TNF-α receptor and protein kinase C activation [61].

In TNF-α-dependent myocardial fibrosis, however, LOX upregulation was noted and found to be due to TNF-α activation of TGF and PI3K signaling [38]. In eosinophilic esophagitis patients, fibroblast-derived TNF-α also induced epithelial LOX expression by activating nuclear factor NF-κB and TGF-β-mediated signaling. LOX upregulation in these patients was indicative of disease complications and fibrostenotic conditions [62]. 

The pro-inflammatory Interleukin 1β (IL-1β) contributes to LOX overexpression in polycystic ovary syndrome (PCOS), with increased LOX and IL-1β levels being observed in granulosa cells and follicular fluid of PCOS patients. In a rat model of PCOS, IL-1β was shown to increase LOX expression via ERK1/2 and JNK, and via the subsequent activation of the transcription factor c-JUN, while inhibition of LOX activity by BAPN improved some of the symptoms [63]. A contrasting IL-1β-mediated downregulation of LOX was reported in several tissues. In human amniotic tissue explants and amnion fibroblasts, IL-1β inhibited LOX via activating p38 and ERK1/2 mitogen-activated protein kinase pathways, resulting in NF-κB phosphorylation as well as GATA3. Subsequently, the activated NF-κB interacted with GATA3 at the NF-κB binding site within LOX promoter inhibiting gene expression [64].

In cerebral aneurysms, prominent degradation of the extracellular matrix and the vascular wall involves reduced LOX expression and elevated IL-1β that, in aortic smooth muscle cells, was confirmed to activate NF-κβ, the inhibition of which restored LOX levels [65]. The IL-1β is a known mediator in the acute inflammatory phase of ligament injury with reduced LOX expression in ligaments [66]. In synovial fibroblasts, the combined effect of elevated TNF-α and IL-1β resulted in reduced LOX expression that, together with increased MMPs, was proposed to impede remodeling and contribute to the poor healing ability of ligaments [67].

Both, IL-1α and IL-4 are involved in ovarian surface epithelial postovulation injury and repair cycles in both pro- and anti-inflammatory mechanisms via different signaling pathways. The anti-inflammatory actions of IL-4 involving SATA6, PI3K and the p38MAPK pathways in these cells were shown to upregulate LOX mRNA levels [68]. In contrast, IL-6 stimulation in osteoblasts was reported to result, via JAK2, Fli1 and Dnmt1, in the downregulation of LOX expression by epigenetic CpG methylation, a mechanism that negatively affects bone matrix formation [69]. In a cohort of keratoconus patients with corneal thinning and structural abnormalities, reduced LOX mRNA and activity were noted in the corneal epithelia that correlated with disease severity. However, in this study, no causality was established between LOX and the parallel increase in IL-6 levels [70]. 

Similarly, the pro-inflammatory Interferon gamma (IFN-γ), active in aortic aneurysm, and arteriosclerotic plaque rupture, were demonstrated to reduce LOX mRNA and activity in rat aortic smooth muscle cells, partly via transcriptional downregulation, and also by reducing the LOX mRNA half-life [71]. Additionally, IFN was shown to also reduce TGF-β-induced LOX levels in cardiac fibroblasts [72]. The mechanistic details of LOX regulation by inflammatory mediators are summarized in Table 2. 

The involvement of LOX in disorders with inflammatory components has been well-established. The role of TNF-α is pleiotropic; it inhibits the functions of LOX in diabetes, osteoporosis and rheumatic arthritis, but also upregulates LOX in myocardial fibrosis and in esophagitis patients. The overall regulatory roles, mechanisms and in vivo significance of IL-1β, IL-1α, IL-4, IL-6 and IFN-γ, while recognized to affect LOX, are less clear at this stage.

## 8. LOX in Steroid Signaling Regulatory Loops

An earlier report identified LOX as part of endocrine-, paracrine- and autocrine-level coordinated regulatory loops in rat ovaries during follicular development. Follicle stimulating hormone (FSH) was shown to be a determinant of activation or inhibition of both LOX mRNA and catalytic activity by local dihydrotestosterone, growth differentiation factor-9 (GDF-9), activin A and TFG-β1 [73]. In primary human ovarian surface epithelial cells, following IL-1α stimulation, expression profiling identified the genes involved in the synthesis of steroid hormones and their receptors, sterols and retinoids, upregulation of 11beta-hydroxysteroid dehydrogenase type 1 (11β-HSD1), suppression of steroidogenic gene 3betaHSD1 and a subset of genes including IL-6, IL-8, NF-κB inhibitor alpha and, notably, LOX as inflammation-associated genes responsive to glucocorticoid regulation [74].

Estradiol (E2) is a positive regulator of LOX gene expression, as confirmed both in the urogenital tissues of mice with accelerated ovarian aging, and in human Ishikawa cells. In these models, as a result of inhibitory tests, it was proposed that TGF-β1 signaling mediates the E2 upregulation of LOX [75]. 

Studies aimed at the mechanisms responsible for fetal membrane rupture and preterm birth (including cortisol regeneration in the amnion and its role in modulating LOX regulation) identified, in human primary amniotic fibroblasts, the induction of components of prostaglandin E2 (PGE2) synthesis, cortisol stimulated 11-hydroxysteroid dehydrogenase 1 (11β-HSD1) and reciprocal inhibition of LOX. The same mechanism was confirmed in human amnion tissue explants. In contrast, LOX inhibition was effectively reversed by glucocorticoid receptor inhibition and a mutation of a negative glucocorticoid LOX promoter element. These results highlighted the role of local cortisol in the amnion in downregulating LOX gene expression through a negative response element in the LOX promoter with deficient LOX functions contributing to fetal membrane rupture [76]. Further details for prostaglandin E2 (PGE2) modulating EP2/EP4 receptor-coupled cAMP/PKA signaling have also emerged, including a feed-forward loop diminishing LOX levels in human amnion fibroblasts during end stage gestation, a mechanism that may similarly contribute to fetal membrane rupture [77]. Table 3 summarizes the LOX regulatory interactions of FHS, estradiol, cortisol and prostaglandin. 

LOX has been known to be subject to glucocorticoid regulation via a negative LOX promoter element. More recent studies emphasized the significance of steroid regulation of LOX and its contribution to fetal membrane rupture.

## 9. Conclusions and Future Directions

The involvement of LOX in regulatory pathways has long been recognized. More recent studies have revealed LOX-associated mechanisms, in which LOX is not only a target of regulatory pathways, but also an active player in signaling events that include LOX-mediated modifications of cell surface receptor functions and mutual regulatory activities in interactions with signaling mediators in processes that contribute to a range of pathological conditions. 

There is, however, some degree of limitation in our current understanding of the more general roles of LOX in regulatory contexts, as the downstream consequences have not yet been fully characterized, and data were obtained in specific cell lines and/or in cell and animal disease models with only a subset of results derived from or confirmed in human in vivo studies.

It is particularly important to fill these knowledge gaps, as LOX has been shown to be a promising target for therapeutic intervention in cancer [3,78,79] and in vascular [80], myocardial [81,82], peritoneal [83] liver [84] and urological disorders [85], in adipose tissue dysfunction with an inflammatory component [86] and in inflammation in Crohns’ disease [87]. In some of these conditions, fibrosis is the pathological endpoint linking aberrant LOX to its ECM cross-linking activity as an attractive target for LOX-inhibitory therapies. In other disorders, including those with inflammatory components, multiple upstream regulatory mechanisms are involved in controlling LOX and, importantly, LOX also contributes to signaling interactions with wide-ranging in vivo downstream effects. In order to realize the full potential of LOX as a therapeutic target with mitigated negative consequences, a more comprehensive understanding of the full spectrum of LOX functions is needed, including the mechanistic details of its involvement in regulatory pathways. 

## Figures and Tables

**Table 1:** Lysyl oxidase (LOX) regulatory and cross-regulatory interactions with signaling pathways.

Signaling Mediators	Interaction/Activity	Signaling Pathways Involved	Refs.
Ang II	Ang II upregulated LOX via EGFR transactivation	EGFR transactivator ADAM17, EGFR/PI3K/AKT, MEK/ERK and SAPK/JNK (lung carcinoma); Oxidative stress-activated p38MAPK (vascular remodeling)	[11,12,16]
EGFR	LOX-controlled modulation of EGFR cell surface availability and EGF activation	Suppressed TGF-β signaling leading to HTRA1/increased MATN2 that traps EGFR at cell surface (tumor progression)	[13]
PDGF	LOX-induced modification/oxidation of cell surface PDGFRβ	Faster turnover of PDGFR-dependent SHP2, AKT1, ERK1/2 (chemotactic response); PDGF/TGF-β1/ERK1/2 (vascular remodeling, myelofibrosis); PDGFRβ/Akt/VEGF (angiogenesis)	[17,18,19,20,22]
VEGF	Mutual positive regulation	LOX-activated AKT via PDGFRβ/increased VEGF (endothelial cells, hepatocellular carcinoma, diabetic neovascularization); VEGF-promoted LOX activity via Akt/ERK/JNK/p38/NF-κB (endothelial angiogenesis)	[22,26,27,28,29,30]
	Cu-related coregulation	VEGF/LOX upregulation by Cu-dependent activation of HIF-1α (angiogenesis); VEGF/LOX expression coordinated with HIF-1α by DAC2	[34]
TGF-β	Coregulation of LOX with ECM substrates	TGF-β/p38MAPK via TGF-β response promoter elements in the LOX and the COL1A1/A2 genes	[37]
	Induction of LOX gene expression	TGF-β and TGF-βR1; PI3K, Smad3, p38-MAPK, JNK, ERK1/2 (fibrosis); Smad2/3 promoted C-JUN/AP-1 (myocardial fibrosis); TGF-β/SMAD3 (preeclampsia); SMAD4 (osteogenesis)	[35,36,38,39,40,41]
	LOX/TGF-β feedback loop	LOX-modulated TGF-β1 regulating myofiber and muscle ECM balance and in inflammatory fibrotic stage (pulmonary fibrosis)	[43,44]
	Direct interaction: LOX-induced oxidative changes altered TGF-β receptor biding	Diminished TGF-β1 induced SMAD3 activation in a cross-talk with PI3K and AKT	[45]
Integrins	LOX-stabilized ECM-mediated regulation	TGF-β pathway activation and a positive feedback for LOX expression	[49,50]
	Stromal stiffness promoted LOX	Activation of integrin β1/GSK-3β/β-catenin (hepatocellular carcinoma) or Rho-Rho kinase myosin light chain axis (osteoarthritis)	[51,53]
	ECM stiffness-driven inflammation, elevated LOX	Involving Rho activator GEF-H1 (lung endothelia)	[52]
	Injury-driven stromal alterations	TGF-β and LOX-dependent activation of integrin β1/pFAK/pAKT (epidermolysis bullosa subtype) or PI3K/AKT (angiogenesis)	[54,55]
	LOX promoted macrophage infiltration	Integrin β1/PYK2 activation via SRC/AKT/YAP1 (macrophages, glioblastoma);	[58]

Ang II: type 2 angiotensin; EGFR: epidermal growth factor receptor LOX: lysyl oxidase; PDGF: platelet derived growth factor; VEGF: vascular endothelial growth factor; TGF-β: transforming growth factor beta; ADAM17: ADAM metallopeptidase domain 17; PI3K: phosphoinositide 3-kinase; AKT: protein kinase B; ERK: extracellular signal-regulated kinase; MEK: mitogen-activated protein kinase kinase/extracellular signal regulated kinase; SAPK: stress activated protein kinase; JNK: Jun N-terminal kinase; p38MAPK: P38 mitogen-activated protein kinase; HTRA1; M: HtrA serine peptidase 1; ATN2: atrophin 2; SHP2: SH2 domain protein tyrosine phosphatase, NF-κB: nuclear factor kappa B; HIF: hypoxia-inducible factor; ECM: extracellular matrix; COL1: type I collagen; AP-1: activator protein-1; GSK3: glycogen synthase kinase 3; GEF: guanine nucleotide exchange factor; FAK: focal adhesion kinase:; PYK2: proline-rich tyrosine kinase 2; YAP: yes-associated protein.

**Table 2:** LOX regulation in inflammatory pathways.

Inflammatory Mediators	Regulatory Activity	Signaling Pathways Involved	Refs.
TNF-α	LOX inhibition in chronic inflammation	TNF-α downregulation via Vegf and Fak (mouse model); miR203-mediated silencing (mesenchymal cells); TNF-α receptor and protein kinase C activation-mediated (endothelial cells)	[59,60,61]
	LOX upregulation	TGF-β/PI3K signaling (myocardial fibrosis); NF-κB/TGF-β-mediated signaling (fibroblast-epithelial interactions, esophagitis)	[38,62]
IL-1β	Induced/inhibited LOX expression	Overexpression via ERK1/2/JNK and c-JUN activation (rat granulosa cells); inhibition by p38 and ERK1/2, NF-κB activation and interaction with GATA3 at the NF-κB binding LOX promoter site (amnion); via IL-1β-activated NF-κβ (aortic smooth muscle cells); IL-1β-mediated inhibition (ligaments)	[63,64,65,66]
IL-4	Pro-/anti-inflammatory activity-related upregulation of LOX	SATA6, PI3K, p38MAPK (ovarian epithelium)	[68]
IL-6	Epigenetic control of LOX expression	Downregulation through JAK2, Fli1 and Dnmt1 (osteoblasts)	[69]
IFN-γ	Pro-inflammatory control of LOX	Downregulation by transcription and mRNA half-life control (aortic smooth muscle cells, cardiac fibroblast)	[71,72]

TNF: tumor necrosis factor; IL: interleukin; IFN: interferon; VEGF: vascular endothelial growth factor; TGF-β: transforming growth factor beta; PI3K: phosphoinositide 3-kinase; ERK: extracellular signal-regulated kinase; JNK: Jun N-terminal kinase; p38MAPK: P38 mitogen-activated protein kinase; NF-κB: Nuclear Factor kappa B; PI3K: phosphatidylinositol-3-kinase FAK: focal adhesion kinase; JAK: Janus kinase; : Flil: Flagellar protein; Dnmt1: DNA (cytosine-5)-methyltransferase 1; LOX: lysyl oxidase.

**Table 3:** Steroid regulation of LOX in ovarian and urogenital cells and tissues and in fetal membrane rupture.

Steroid Hormones	Regulatory Activity	Signaling Pathways Involved	Refs.
Follicle stimulating hormone	FHS activation/inhibition of LOX mRNA/activity	Local dihydrotestosterone, GDF-9, activin A, and TFG-β1 (rat ovaries)	[73]
Estradiol (E2)	Intersection with TGF-β1/LOX	TGF-β- mediated E2 upregulation of LOX gene expression (mouse urogenital tissue, Ishikawa cells)	[75]
Cortisol	LOX inhibition by cortisol induced PGE2 and 11β-HSD1	Regulation via the negative steroid LOX promoter element (amniotic fibroblasts and tissue, fetal membrane rupture)	[76]
Prostaglandin E2 (PGE2)	PGE2-induced feed-forward loop targeting LOX	EP2/EP4 receptor-coupled cAMP/PKA pathway (amniotic fibroblasts, fetal membrane rupture)	[77]

FHS: Follicle stimulating hormone; E2: estradiol; PGE2: prostaglandin E2; 11β-HSD: 11beta-hydroxysteroid dehydrogenase; EP2& 4: prostaglandin E2& 4 receptors; cAMP: cyclic adenosine monophosphate; PKA: protein kinase A; GDF: growth and differentiation factor; TNF: tumor necrosis factor; IL: interleukin; IFN: interferon; VEGF: vascular endothelial growth factor; TGF-β: transforming growth factor beta; PI3K: phosphoinositide 3-kinase; ERK: extracellular signal-regulated kinase; JNK: Jun N-Terminal Kinase; p38MAPK: P38 mitogen-activated protein kinase; NF-κB: Nuclear Factor kappa B; PI3K: phosphatidylinositol-3-kinase FAK: focal adhesion kinase; JAK: Janus kinase; : Flil: flagellar protein; Dnmt1: DNA (cytosine-5)-methyltransferase 1; LOX: lysyl oxidase.
